# Lanthanum from a Modified Clay Used in Eutrophication Control Is Bioavailable to the Marbled Crayfish (*Procambarus fallax* f. *virginalis*)

**DOI:** 10.1371/journal.pone.0102410

**Published:** 2014-07-28

**Authors:** Frank van Oosterhout, Eyerusalem Goitom, Ivo Roessink, Miquel Lürling

**Affiliations:** 1 Aquatic Ecology & Water Quality Management Group, Department of Environmental Sciences, Wageningen University, Wageningen, The Netherlands; 2 Alterra, Wageningen UR, Wageningen, The Netherlands; 3 Department of Aquatic Ecology, Netherlands Institute of Ecology (NIOO-KNAW), Wageningen, The Netherlands; University of Shiga Prefecture, Japan

## Abstract

To mitigate eutrophication in fresh standing waters the focus is on phosphorus (P) control, i.e. on P inflows to a lake as well as a lake's sediment as internal P source. The in-lake application of the lanthanum (La) modified clays – i.e. La modified bentonite (Phoslock) or La modified kaolinite, aim at dephosphatising the water column and at reducing the release of P from a lake's sediment. Application of these clays raises the question whether La from these clays can become bioavailable to biota. We investigated the bioavailability of La from Phoslock in a controlled parallel groups experiment in which we measured the La in carapace, gills, ovaries, hepatopancreas and abdominal muscle after 0, 14 and 28 days of exposure to Phoslock. Expressing the treatment effect as the difference of the median concentration between the two treatment groups (Phoslock minus control group) yield the following effects, the plus sign (+) indicating an increase, concentrations in µg g^−1^ dry weight: Day 14: carapace +10.5 µg g^−1^, gills +112 µg g^−1^, ovaries +2.6 µg g^−1^, hepatopancreas +32.9 µg g^−1^ and abodminal muscle +3.2 µg g^−1^. Day 28: carapace +17.9 µg g^−1^; gills +182 µg g^−1^; ovaries +2.2 µg g^−1^; hepatopancreas +41.9 µg g^−1^ and abodminal muscle +7.6 µg g^−1^, all effects were statistically significant. As La from Phoslock is bio-available to and taken up by the marbled crayfishes (*Procambarus fallax* f. *virginalis*), we advocate that the application of in-lake chemical water treatments to mitigate eutrophication should be accompanied by a thorough study on potential side effects.

## Introduction

Eutrophication, i.e. over-enrichment of surface waters with nutrients, is a worldwide water quality issue [1], which often results in massive algal blooms. In freshwater systems the majority of these blooms are formed by cyanobacteria [2], which are a threat to human health and husbandry [3], [4], [5], [6]. Such blooms are in conflict with the good water quality demanded by legislation (European Water Framework and Bathing Water Directives[7], [8], which makes controlling eutrophication and thereby preventing cyanobacteria nuisance a key challenge to water quality managers.

Mitigation of eutrophication principally focuses on phosphorus (P) control [9], [10]. In this, reducing the external inputs of P to a lake is an obvious method to mediate recovery from eutrophication, but it may take many years for a lake to recover after the external loading is reduced [11], [12]. This because during periods of high external P loading, lake sediments have accumulated substantial amounts of P. After external loading has ceased, release of P from the sediment back into the water column impedes water quality improvement [11]. Not taking a lake's internal P loading into account is identified as one of the major causes for failing lake restoration [13]. Removal (dredging) of nutrient rich sediments is a straightforward measure in tackling the legacy P; however, dredging is relatively expensive. Targeting the release of P from the sediments with a P-fixative is a more economic option [14]. Regarding the latter, new methods are the application of La modified clays with a high P binding capacity – e.g. the La modified bentonite (Phoslock) [15] and LaCl_3_-modified kaolinite [16], which aim at both dephosphatisation of the water column as well as capping of the sediment to prevent release of P.

In recent years, the La modified bentonite (LMB) Phoslock has been applied to at least 20 lakes (http://www.phoslock.eu/en/applications/case-studies/; last accessed June 8^th^, 2014), whereas the manufacturer speaks of “more than 100 applications have been completed in over 20 countries” (http://www.phoslock.eu/en/phoslock/ecotox/; last accessed June 8^th^, 2014).

Although the application of LMB is reported as a promising method in controlling eutrophication [17], [18], [19], [20], [21], [22], there are concerns regarding the environmental safety of lanthanum modified clays. Depending on chemical water composition and concentration of dissolved or free ion forms of La, this rare earth metal can be toxic to aquatic organisms [18], [19], [23], [24]. To decrease its toxicity, La is incorporated in a bentonite clay [21]. A review of 16 case study lakes confirms that release of filterable La to the water column following the application of the LMB [25]. Much of the environmental safety of the LMB is derived from the fact that La is stored in the bentonite matrix and the extreme low solubility (*K*_sp_ = 10^−24.7^ to 10^−25.7^ mol^2^ l^−2^) [26], [27] of the La-phosphate complex (Rhabdophane), from which it is deducted that La will not be bioavailable after an application of LMB.

The safety of LMB is described in a ‘full public report’ [24]. However, the bioavailability of La is not tested [24], although there is some evidence in literature that La could be taken up or stored in biota, e.g. in blue mussels (*Mytilus edulis*) [28] and in duckweeds (*Spirodela polyrhiza*), Cladocerans (*Daphnia magna*), goldfish (*Carassius auratus L.*) and shellfish (*Bellamya aeruginosa*) [29]. Since LMB is applied to a water body as slurry that settles as a thin layer on the sediment, it is expected that especially bottom-dwelling organisms may be exposed to relatively high concentrations of the modified clay and potentially to La. Therefore, especially research on crayfish is highly relevant as these larger, benthic crustacean species might be particularly vulnerable to compounds on or in the sediments, because of their close contact with sediment particles [30]. Moreover, crayfish have been proposed and used as bioindicator for the aquatic contamination by heavy metals [31], [32], [33], [34]. To test whether exposure of bottom-dwelling organisms to the modified clay may indeed result in an (enhanced) uptake of La and possibly negative effects on the biota, experiments with laboratory-cultured freshwater marbled crayfish (*Procambarus fallax* f. *virginalis*; [35]) were done.

This paper presents the results from a parallel groups experiment in which the La content of unexposed (controls) and LMB exposed marbled crayfish is compared after 0, 14, and 28 days exposure. To exclude potential sorption of La to the outside of the animal and to address possible tissue specific distribution, not the whole animal, but specific tissue of the gills, hepatopancreas, carapace, ovaries and abdominal muscle were analysed. For each tissue we test the hypothesis that there is no difference in La concentration (µg g^−1^) between the controls and exposed animals after 0, 14, and 28 days.

## Methods

### Experimental design

Under Dutch law, experiments with freshwater crayfish do not require approval of the Animal Experiment Committee (in Dutch: Dier Experiment Commissie, DEC). Animal welfare was monitored daily and at termination of the test period animals were culled quick and humanely to avoid any unnecessary suffering.

**Figure 1:**
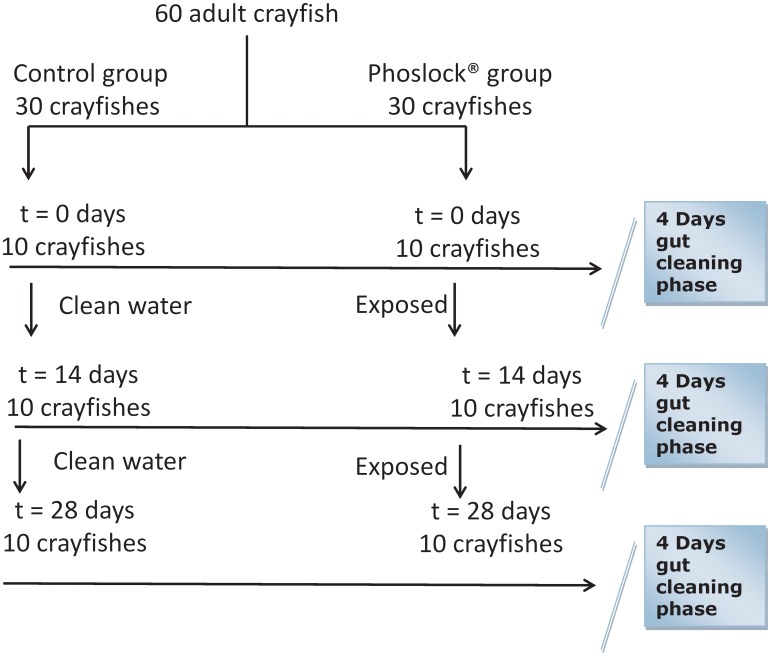
Experimental design, see text.

Sixty adult marbled crayfishes (*Procambarus fallax* f. *virginalis* [35]) obtained from stock culture (Alterra, Wageningen UR) were randomly assigned to either control or LMB treatment group (Fig. 1). The LMB used was the commercially available lanthanum modified bentonite Phoslock, which was obtained from Phoslock Europe GmbH (Ottersberg, Germany). Randomisation was stratified according to body length (from tip of rostrum to tip of telson). The experiment was conducted according to a parallel group design, i.e. one control and one treatment with a 7 day run-in phase, followed by either a 14 or 28 day exposure phase (Fig. 1). After run-in, the LMB treated group received approximately 1000 mg l^−1^ LMB per experimental unit, while the controls were kept without LMB. The LMB dosage was based on practical feasibility – e.g. added LMB in combination with aeration yields turbid experimental units which impairs visual inspection of the crayfish. From each treatment group 10 animals were sampled at the end of run-in (day 0), day 14, and day 28, respectively. All sampled animals were individually kept 4 days in clean containers with copper free water devoid of LMB to empty their gut (depuration) before further processing. Dutch tap water contains copper which can result in toxic effects on especially crustaceans - e.g. [36], [37], hence we used copper free water. Anticipating possible deaths, we chose to enter the maximum number (60 crayfish) practically feasible into the experiment. Of these, the tissues of 36 crayfish were analyzed for La- hence from each treatment group the tissues of 6 animals were analysed as sampled at the end of run-in (day 0), day 14 and day 28 respectively.

### Experimental set-up

Individual crayfish were placed in 13.5×11.0×9.0 cm (1.3 l) transparent plastic containers and kept under climatized conditions (20°C, semi dark, i.e. 12 hours indirect diffuse light, 12 hours dark, constant aeration) in 1 litre copper free tap water (pH = 7.5 and EC = 194.5 µs cm^−1^) for 7 days (run-in) prior to the experiment. The containers were closed with a transparent lit. To facilitate both adequate mixing and oxygen saturation each container was aerated through a small hose fitted through the lit. To avoid toxicity due to accumulation of waste products like ammonia, each container was refreshed weekly: copper free tap water for controls or a new 1000 mg l^−1^ LMB suspension in copper free tap water. The animals were fed two times per week (approximately 12 mg per crayfish) commercial fish food pellet (a product of Trouw Nutrition Nederland B.V.). The general health status of the crayfish with respect to death, moulting or egg bearing was observed daily.

After depuration the animals were euthanized by burying them in ice for 30 minutes. To give the tissues a better texture for dissecting, the crayfish were immersed in hot tap water for 2 minutes after euthanisation. To remove possibly attached LMB from the outside of the carapace the crayfishes were rinsed two times with copper free water. Prior to dissection, the total body weight and body length of all animals was measured. The specimens were dissected to obtain tissues samples from carapace, gills, ovaries, hepatopancreas and abdominal muscle. Tissue samples were individually put in 30 ml plastic containers, closed with a lid, weighed and stored at (−20°C). Subsequently, the tissues were freeze- dried (−60°C) for 24 hrs, crushed (using pestle and mortar), where after approximately 20 mg per tissue sample was destructed with the combination of Ultrex HNO_3_ (65%) and H_2_O_2_ (30%) [38]. The La concentration in the destruates was determined by ICP-MS at the Chemical- Biological Soil Laboratory of the Department of Soil Sciences (Wageningen University Research Centre). As a control for the origin of La in the tissues we tested 3 fish food samples, processed as described above. Four copper free water samples were analysed for La.

### Water quality variables

Temperature (°C), dissolved oxygen (mg l^−1^), pH, electric conductivity (EC, µS cm^−1^) and turbidity (NTU) were measured using Oxy Guard Handy Gamma oxygen meter, WTW 320 pH meter, Cond 315i WTW conductivity meter, and HACH 2100P turbidity meter, respectively. Except for week 4 - in which they were assessed once, these measurements were taken twice per week.

As ammonia is known to be toxic to crayfish at concentrations above 50 mg l^−1^ [39], we monitored the ammonia concentration in our experiment. The ammonia concentration (mg l^−1^) was determined in a random subset of 10 experimental units per treatment per week. The filterable (0.45 µm, Whatman NC45) lanthanum concentration was determined in a random subset of 4 experimental units per treatment per week. The samples were taken shortly before refreshing. The filtered (0.45 µm, Whatman NC45) samples were analysed for ammonia concentration using Skalar continuous flow analyzer following the Dutch standard protocols [40], [41], [42], filterable La concentration was determined by ICP-MS at the Chemical- Biological Soil Laboratory of the Department of Soil Sciences (Wageningen University Research Centre). The ICP-MS level of detection for La in the matrix (HNO3) is 0.2 µg l-1, in water it is 0.02 µg l-1. Tissue concentrations are expressed as (µg g^−1^) dry weight (DW).

Cross contamination between, as well as within the groups was prevented by following strict operational procedures. Controls were always handled or measured before LMB treated experimental units, after replenishing or measurements the container was directly closed with its lit, the probes were rinsed with copper free and demi water between measurements. The depuration procedure was followed to avoid possible contaminations of tissue samples through gut content during subsequent dissection. During the euthanisation and hot water procedure, cross contamination was prevented by first putting individual animals in small sealed plastic bag. Throughout dissecting, crushing and destruction control samples were processed before LMB treated samples, using clean dissection equipment for each animal, and cleaning the pestle and mortar with demi water and 80% ethanol using tissue paper between samples.

### Data analysis

For the water quality variables temperature, dissolved oxygen concentration, pH, EC, NTU, ammonia concentration and filterable lanthanum we present descriptive statistics only. As the number of deaths and egg bearing animals in both control and LMB treated groups numerically came out quite the same we did not formally test these. The number of moults was compered by chi-square test. As body length (mm), body weight (g) and their growths per week as well as the tissue lanthanum concentrations were non-normally distributed we compared the treatment groups by Mann-Whitney U test, we report median and min, max values in conjunction with the Mann-Whitney U test. The lanthanum concentration in the selected tissues of the crayfish are reported as median (min-max) (µg g^−1^) dry weight (DW) together with the test results. For convenience the results are also presented as means and standard deviations in a graph Statistics were done in SigmaPlot 11.0. All statistical tests were conducted with a level of significance α = 0.05. Unless stated otherwise the number of observations (*n*) for water quality variables is 119 and 120 in the control respectively LMB group.

## Results

Regarding water temperature, dissolved oxygen concentration, pH, EC, and ammonia, no relevant differences were observed between the control and LMB treated groups (Table 1). NTU in the LMB treated group was much higher than in the control group (Table 1).

**Table 1:** Descriptive statistics for temperature (T,°C), dissolved oxygen (DO_2_, mg l^−1^), pH, conductivity (EC, µs cm^−1^), Turbidity (NTU), ammonium (NH_4_-N, mg l^−1^) and filterable lanthanum (La, µg l^−1^) for both control (Control) and lanthanum modified bentonite Phoslock groups (Phoslock) throughout the experiment; except for pH, mean and standard deviations (SD) are given for all variables, for pH median, minimum (min) - maximum (max) values are given.

	Control		Phoslock	
	N = 119		N = 120	
	mean	SD	mean	SD
T	20.0	(0.2)	20.0	(0.2)
DO_2_	8.8	(0.3)	8.8	(0.2)
pH median(min-max)	8.1	7.4–8.2	8.0	7.4–8.2
EC	181.0	(32.0)	183.0	(24.7)
NTU	0.8	(0.4)	100.2	(114.3)
	N = 40		N = 40	
Ammonia	0.1	(0.4)	0.9	(1.2)
	N = 16		N = 16	
Filterable lanthanum	0.04	(0.03)	16.80	(32.95)

Survival in the LMB treatment groups was 100%, in the control group two crayfish died during run-in (which were replaced) and one during week 1 (which was not replaced) The number of moulting (Χ^2^_df = 1_ = 1.4, p = 0.2) and egg bearing crayfish were similar amongst the two treatment groups. In the control group (N = 19) 14 moulted and 4 bore egss during the course of 28 days experiment. In the LMB treated group (N = 20) 10 animals moulted and 5 bore eggs.

No statistically significant differences were observed between the groups with respect to body length, weight and their growth. Median (25%–75% percentiles) of body length at the start were 54.8 mm (48.3–58.0 mm) and 52.9 mm (51.1–57.2 mm) in the control respectively LMB group (U = 181, p = 0.6), median (25%–75% percentiles) of body weight at the start were 3.9 g (2.6–4.8 g) g respectively 3.6 g (3.1–4.3 g) (U = 193, p = 0.9). Median (25%–75% percentiles) growth in body length were 0.5 mm week^−1^ (0.2–1.1 mm.week^−1^) and 0.6 mm week^−1^ (0.2–1.3 mm week^−1^ in the control respectively LMB treated groups (U = 174, p = 0.5). In both groups, median (25%–75% percentiles) growth in body weight were 0.1 g week^−1^ (0.0–0.2 gweek^−1^) (U = 190, p = 0.8).

The copper free tap water used in both treatments contained 0.04 µg l^−1^ (SD = 0.03, *n* = 4) La. The fish food used in both treatment groups contained 58.3 (SD = 56.0, *n* = 3) µg g^−1^ dry weight La. The LMB treated group received 1.0038 g l^−1^ (SD = 0.0030, *n* = 60) LMB per experimental unit per week. Filterable La concentration in the water of the control group was 0.04 µg l^−1^ (SD = 0.03, *n* = 16), while in the LMB group it was 16.80 µg l^−1^ (SD = 32.95, *n* = 16) (Table 1).

In all tissues samped at day 0– before treatment, we found a background concentration of La in both treatment groups (Table 2), which was slightly higher in the control group than in the LMB treated group. The difference of the median La concentration between the two treatment groups (LMB - control group) at day 0 were: carapace −0.4 µg g^−1^; gills −0.3 µg g^−1^; ovaries −1.0 µg g^−1^; hepatopancreas −0.1 µg g^−1^ and abodminal muscle −0.1 µg g^−1^. These differences reached statistical significance between the treatment groups for carapace, gills and abdominal muscle (Table 2).

**Table 2:** Median, minimum (min), maximum (max) of lanthanum concentrations (µg g^−1^ DW) in carapace, gills, ovaries, hepatopancreas and muscle tissue of crayfish at day 0, 14, and 24; U =  Mann-whitney U statistic, the associated N is given in the header; ***p*** **=** ***p*** **-value of the test.**

		Control N = 6		Phoslock N = 6			
	Day	median	(min - max)	median	(min - max)	U	*p*
Carapace	0	0.4	(0.2–0.6)	0.0	(0.0–0.1)	0	0.002
	14	0.1	(0.0–6.6)	10.6	(2.1–35.1)	1	0.004
	28	0.1	(0.0–0.2)	18.0	(8.0–23.8)	0	0.002
Gills	0	0.5	(0.5–1.2)	0.2	(0.1–0.6)	4	0.026
	14	2.1	(0.4–7.6)	114.0	(51.6–600.2)	0	0.002
	28	1.5	(0.6–3.5)	183.5	(125.2–962.0)	0	0.002
Ovaries	0	1.1	(0.3–1.4)	0.1	(0.0–1.5)	8	0.13
	14	0.1	(0.0–1.5)	2.7	(1.5–4.3)	0	0.002
	28	0.1	(0.0–0.8)	2.3	(0.8–7.7)	0	0.002
Hepatopancreas	0	0.2	(0.1–0.9)	0.1	(0.0–0.2)	8	0.13
	14	0.8	(0.1–2.7)	33.3	(4.7–200.0)	0	0.002
	28	0.2	(0.1–0.9)	44.1	(19.2–306.3)	0	0.002
Abdomial muscle	0	0.1	(0.0–0.1)	0.0	(0.0–0.1)	1	0.004
	14	0.1	(0.0–0.4)	2.3	(1.1–6.2)	0	0.002
	28	0.0	(0.0–0.1)	7.6	(4.4–13.6)	0	0.002

**Figure 2:**
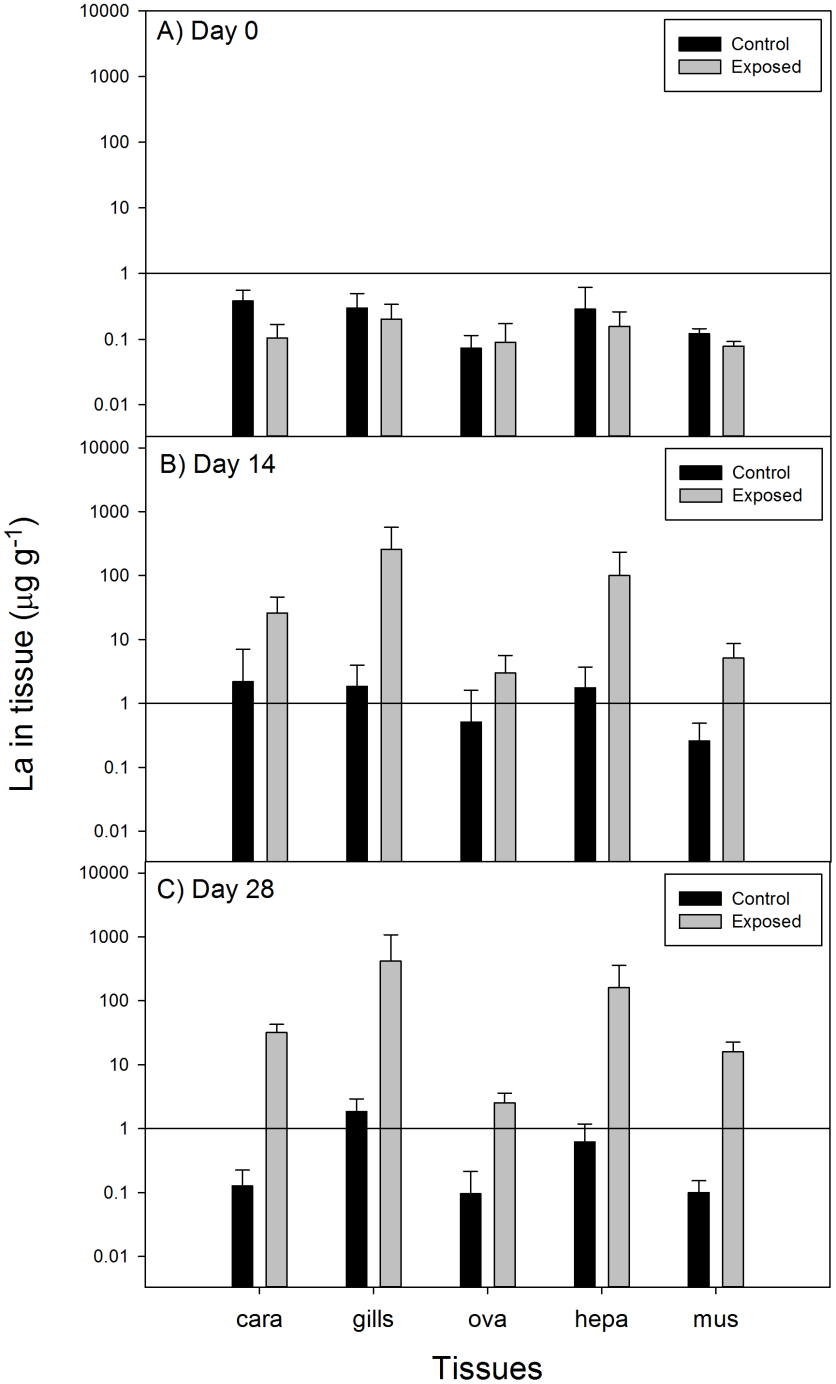
Lanthanum concentration (µg g^−1^ DW) in carapace (cara), gills (gills), ovaries (ova), hepatopancreas (hepa) and muscle (mus) tissue of crayfish at day 0, 14, and 28; bars indicate mean, whiskers indicate standard error, all *n* = 6.

After 14 and 28 days of the experiment, no relevant increase was observed in the control group (Tabel 2). In the LMB treated group a significant increase in the lanthanum concentration per DW was found in all tissues after both 14 and 28 days exposure (Fig. 2, Table 2). The differences between the two treatment groups were statistically significant between treatment groups for all five tissues (Table 2). Expressing the treatment effect as the difference of the median concentration between the two treatment groups (LMB minus control group) yield the following effects, the plus sign (+) indicating an increase in the La concentration (µg g^−1^ DW):

Day 14: carapace +10.5 µg g^−1^ DW; gills +112 µg g^−1^ DW; ovaries +2.6 µg g^−1^ DW; hepatopancreas +32.9 µg g^−1^ DW and abodminal muscle +3.2 µg g^−1^ DW.

Day 28: carapace +17.9 µg g^−1^ DW; gills +182 µg g^−1^ DW; ovaries +2.2 µg g^−1^ DW; hepatopancreas +41.9 µg g^−1^ DW and abodminal muscle +7.6 µg g^−1^ DW.

## Discussion

After exposure to LMB the La concentration in the crayfish tissues of the LMB treated group had gone up several orders of magnitude, e.g. as compared to the control group the lowest increase was 23 fold in abdominal muscle after 14 days exposure, while a 122 fold increase was observed in the gills after 28 days. Hence, we consider the observed marginal difference between the two groups at the end of the run-in – before treatment, of little meaning. The observed elevated La concentrations in the gills and carapace may – at least partly, be due to LMB particles attach from the outside. However, this is not the case for ovaries, hepatopancreas and abodminal muscle. Hence the increase in La concentration in the ovaries, hepatopancreas and abdominal muscle of the crayfish after exposure to LMB straightforwardly shows that La from the LMB is bioavailable and taken up by the crayfish. Therefore, we reject the hypothesis that exposure of the crayfish to this LMB does not change the La content of the tissues we selected.

Although one specimen died in the controls, none of the exposed crayfish died during the experiment, hence the observed increased La concentrations in the five tissues do not result in acute lethal toxic effects. This, however, does not exclude possible chronic effects, e.g. as measurable by reduced offspring or less fertile offspring, as these endpoints were not included in our experiment. Nonetheless, based on the number of moulting, egg bearing crayfish and observed growth with no significant difference between the treatment groups, we consider all animals to have developed normally and equally among the control and LMB treated groups during our experiment.

The temperature, dissolved oxygen, pH and EC in both treatments treatment groups encompass the ranges where the crayfish can be best cultured [43], [44], [45]. The higher EC, NTU, ammonia and filterable lanthanum concentrations in the water of the LMB group is due to the presence of LMB [22], [46]. The ammonia concentration was well below the range at which toxic effects are to be expected [39]. The NTU is elevated because LMB – which is a fine grained betonite, is resuspended due to the combination of the aeration of the test systems and the activity of the crayfish - hence in the controls the NTU is not elevated. During an actual field application the LMB is applied as a suspension to the water surface – i.e. from a barge through a spray manifold. While its settling rate depends on local circumstances, the application of the LMB elevates the NTU for some time. The LMB settles on the sediment to form a 1–3 mm thick chemical barrier for FRP [47], where wind induced water movements or bioturbation are expected to be able to resuspend the LMB. Although one may expect some effects of the elevated NTU on survival, moulting, egg bearing or growth, this was not observed in our experiment.

The background concentration of rare earths such as La is generally assumed to be fall well below detection limits (which is 0.02 µg l^−1^ for filterable La in samples). Our results show there is detectable La in both the copper free water and fish food we used. One may argue that the 0.04 µg l^−1^ La in the copper free water is fairly low; this is not the case for the 58.3 µg g^−1^ La in the fish food. Rare earth metals are applied as micro fertilizers to promote seed germination, stimulate the growth of roots, increase the content of chlorophyll and enhance the resistance of crops [48], [49], which may account for at least part of the La found in the fish food. Thus, the La found in the tissues of the control group and part of the La we found in the LMB treated group originates from both copper free tap water and fish food. The fact that there is a detectable La concentration in the tissues of the crayfish - before treatment, is already an indication of the bioavailability of La to the crayfish. Similarly, in carps (*Cyprinus carpio*) background concentrations of La were 0.01 µg g^−1^ in muscle tissue, 0.02 µg g^−1^ in bone and 1.04 µg g^−1^ in liver tissue [50]. Also Qiang et al. [51] found background La concentrations of 0.04 µg g^−1^ in muscle tissue of carps, 0.14 µg g^−1^ in bone and 0.12 µg g^−1^ in organ tissues. Livers of rainbow trout (*Oncorhynchus mykiss*) had background La concentrations between 0.1 and 0.55 µg g^−1^ [52].

While externally attachment of LMB particles can be excluded as possible route for La into the ovaries, hepatopancreas and abodminal muscle, the increased La concentration is due to the ingestion of the LMB – which may run through attachment of the LMB to food particles. As ingested food goes from the stomach directly to hepatopancreas (not just producing enzymes delivered to the gut in crayfish), the La concentration measured in this tissue can be also partially influenced by ingestion of the LMB. Hence clear physiological isolation is true just for ovaries and muscle. Using as input for chemical equilibrium modelling (Cheaqs Pro; [53]) the hardness (3 mmol CaCO_3_) and pH (8.0) of the used water, and a 5% La content in the LMB - assuming all La will be dispersed in the water, yields that maximally 0.01% of the La would be dissolved as LaCO_3_^+^, and 99.99% would precipitate as La_2_(CO_3_)_3(s)_. From which we infer that the or uptake of La in dissolved phase after leaching from the LMB without ingestion is unlikely in our experiment. However, this is not the case after field applications. I.e. Spears et al. [25] describe that the filterable La concentrations in water overlaying the sediments of 16 lakes is elevated for 3–12 months after the LMB application [25]. Hence these routes quite likely also apply to gills and carapace in addition to possible attachment of LMB particles. Aquatic invertebrates may take up dissolved trace metals into their body through their permeable body surface [54], for which the gills are one of the pathways [55], [56] and from the diet [57]. These studies also show there are distinct differences in the accumulation of heavy metals between tissues – e.g. copper, nikkel and cadmium in hepatopancreas, gills, carapace, abdominal muscle et cetera. [31], [32]. This, however, does not hold for all metals – e.g. while zinc was found to accumulate in the hepatopancreas, iron was found to be evenly distributed over the hepatopancreas, exosceleton, abdominal muscle, digestive gut and viscera [33]. Meanwhile, magnesium and manganese do not always accumulate according to the pattern observed for other metals - e.g. Mg concentration was highest in the exosceleton, while that of Mn was highest in the digestive gut [33]. While the crustacean hepatopancreas has a function in food absorption and secretion of digestive enzymes [58], it is also identified as the principal site for the accumulation of metals ions [59]. Part of the La concentration found in the gills and carapace may be due to direct sorbtion of La from the surrounding water.

The relative high concentration of La in the gills of the LMB-group indicate that direct uptake of La through the gills is a possible path. This path is also supported by literature [54], [55]. When carps were exposed for 45 days to 0.5 mg l^−1^ lanthanumnitrate, especially in gills and internal organs La concentrations were elevated, but also in muscle tissue and the skeleton [51], this indicates that La can be taken up and incorporated in fish tissue. Support for the bioavailability of La from the LMB is obtained from two applications in Lake Okareka (New Zealand). After the application of LMB in 2006, elevated La concentrations in livers of trouts were observed [52].

Incorporation in clay minerals, not only provides ballast required for La to reach the prime target site, which is the bottom sediment, but also has been suggested to overcome the toxicity of La to some aquatic organisms [21] and to dramatically reduce the availability of its free form [60]. However, LMB leachate in synthetic soft water had an effect on the tiger prawn *Macrobrachium* sp. with a 14 day LC50 of 800 mg l^−1^ and a 21 day LC50 of 700 mg l^−1^ [60]. This might point to toxicity exerted by the La^3+^ ion [61] of which the concentration will be higher in soft water than in hard water. Consequently, propositions as “with the lanthanum bound in the substrate, the lanthanum phosphate complex is effectively immobilised” [15] and “…when appropriately substituted all La is contained within the clay matrix” [62], should be approached critically because it is the chemical characteristics of the water and conditions at the water sediment interface that will determine liberation of lanthanum from the clay matrix and its speciation. Nonetheless, in laboratory tests using non-soft waters no adverse effects of LMB on most aquatic biota has been found so far (e.g., [60], [63], [64], [65]), but the population growth of the rotifer *Brachionus calyciflorus* was reduced at LMB concentrations of 0.2 g l^−1^ and higher [22].

So far, the LMB has been applied to at least 20 lakes (http://www.phoslock.eu/en/applications/case-studies/; last accessed June 8th, 2014). Actual dosage applied are between 60 g m^−2^ (Lake Okareka, New Zealand) and 667 g m^−2^ (Lake Kleiner See, Germany) [25]. As recommended by its manufacturers, the LMB is applied to bind all P present – based on the total P content of the water column and the top cm's of the sediment, which indicates that actual field dosing can even be higher than the 667 g m^−2^ as applied in Lake Kleiner See. Hence the LMB dosage used in our experiment - approximately 1 g l^−1^ or 67.3 g m^−2^ is quite low compared to dosing at field applications. After a field application the LMB settles on top of the sediment where resuspension and bioturbation – e.g. as described by Reitzel et al. [66], will mix the LMB with the sediment. The LBM will remain at high concentrations on top of and in the upper layer of the sediment. In as far as the LMB dosage used in our experiment is low, it accounts for possible lower LMB concentration after a field application due to possible mixing of the LMB with the sediment. However, from these field applications effects on non-target, bottom dwelling organisms have not been reported yet. Hence, there is a clear need for long-term studies evaluating the effects of the application of LMB products such as Phoslock on bioindicator organisms such as crayfish.

Here, we have shown that La concentrations were elevated strongly in crayfish tissues of specimens exposed to the LMB. Inasmuch as La tissue concentrations seemed to increase over time, longer term exposure experiments could be advised. In general accumulation of metals in crayfish tissues appears dose- and time-dependent and could be influenced by depuration [67], making longer term studies crucial in evaluating potential negative effects on the population and community level.

In analogy to the debate on ocean iron fertilization [68], we advocate that the use of LMB products like Phoslock in lakes to restore them should be accompanied by a thorough study on potential side effects.

## Conclusions

Our laboratory study shows that lanthanum from Phoslock is bio-available to and taken up by the marbled crayfishes (*Procambarus fallax* f. virginalis). We advocate that the application of in-lake chemical water treatments to mitigate eutrophication should be accompanied by a thorough study on potential side effects.
